# Supplementary material for: The Development of a Predictive Model for Postoperative Renal Function in Living Kidney-Transplant Donors
Source: J Clin Med. 2024 Nov 23;13(23):7090. doi: 10.3390/jcm13237090 (PMC11641957; doi:10.3390/jcm13237090)
Supplement: Supplementary file 1 [file jcm-13-07090-s001.zip › jcm-3303482-supplementary.pdf]

Table S1. Evaluation of split real function by CT volumetry

	Left kidney	Right kidney	*p*-value
Kidney volume, mL	152.8 ± 30.5	143.5 ± 26.1	< 0.001
Kidney volume rate, %	51.5 ± 2.8	48.5 ± 2.8	< 0.001
Split renal function, mL/min/1.73 m^2	38.8 ± 7.0	36.5 ± 6.3	< 0.001

Data, mean ± SD. The kidney volume ratio is the ratio of the unilateral kidney volume to the total kidney volume.

Table S2. Multiple regression analysis for functional adaptation rate using patient background

Predictor	Estimate	SE	t-value	*p*-value
Sex (male)	–1.319366	0.739063	–1.79	0.0752
Age	–0.317755	0.073997	–4.29	< .0001*
Pre-SRF	–0.884644	0.117974	–7.50	< .0001*
HT (+)	–0.051517	0.843588	–0.06	0.9513
DM (+)	0.7697737	2.251801	0.34	0.7327
HUA (+)	–2.821464	1.276367	–2.21	0.0278*

Estimate, parameter estimate; SE, standard error; Age, donor age; Pre-SRF, preoperative split renal function; HT, history of hypertension; DM, history of diabetes mellitus; HUA, history of hyperuricemia

Table S3. Multiple regression analysis for postoperative renal function using patient background

Predictor	Estimate	SE	t-value	*p*-value
Sex (male)	–0.635526	0.345778	–1.84	0.0675
Age	–0.117598	0.034131	–3.45	0.0007*
Pre-SRF	0.9286009	0.055878	16.62	< .0001*
HT (+)	0.0702639	0.409296	0.17	0.8639
DM (+)	0.2940406	0.925846	0.32	0.7511
HUA (+)	–1.725535	0.652455	–2.64	0.0088*

Estimate, parameter estimate; SE, standard error; Age, donor age; Pre-SRF, preoperative split renal function; HT, history of hypertension; DM, history of diabetes mellitus; HUA, history of hyperuricemia

Table S4. Multiple regression analysis including an interaction term for postoperative renal function, incorporating preoperative split renal function, donor age, and a history of hyperuricemia

**Predictor**	**Estimate**	**SE**	**t-value**	***p*-value**
Age	0.4523229	0.206911	2.19	0.0300*
Pre-SRF	1.5173195	0.289214	5.25	< .0001*
HUA (+)	–0.992582	7.246609	–0.14	0.8912
Age × pre-SRF	–0.013005	0.004155	–3.13	0.0020*
Age × HUA	–0.07972	0.161343	–0.49	0.6218
Pre-SRF × HUA	0.1963504	0.184056	1.07	0.2873

Estimate, parameter estimate; SE, standard error; Age, donor age; Pre-SRF, preoperative split renal function; HUA, history of hyperuricemia

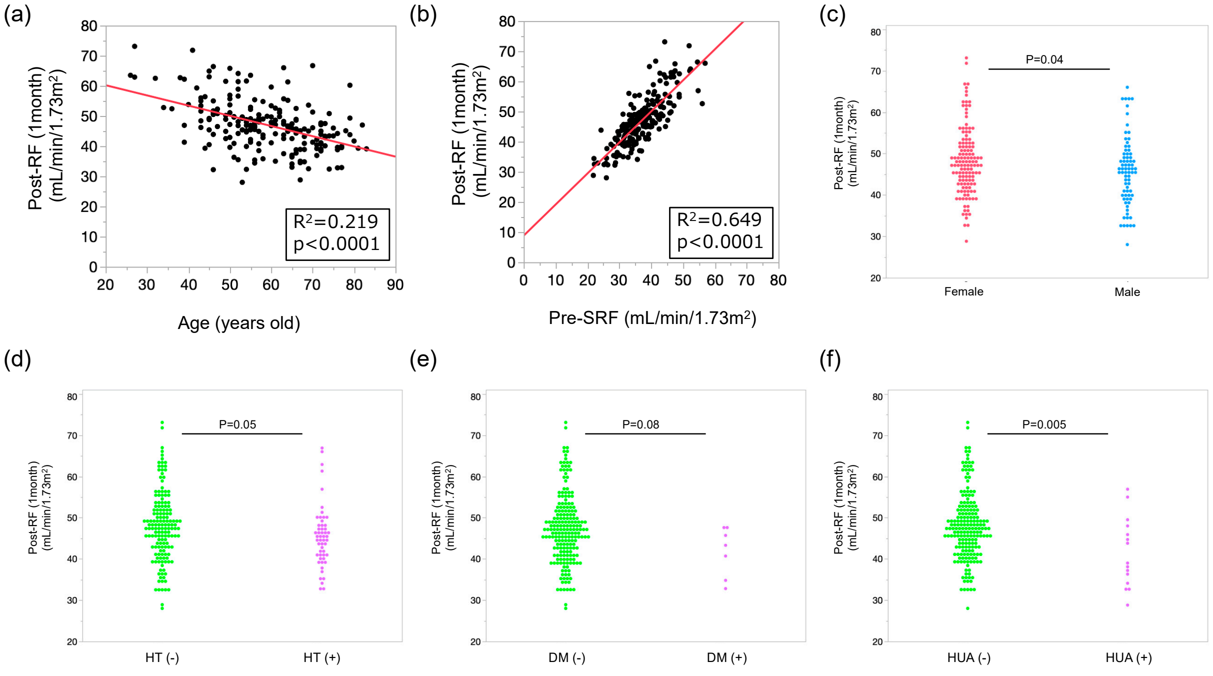

Figure S1. Correlation between patient background and functional adaptation rate in the development cohort

(a, b) Scatterplots of postoperative renal function and (a) preoperative split renal function and (b) age (c–f) Bee swarm plots of postoperative renal function for (c) sex, (d) a history of hypertension, (e) a history of diabetes, and (f) a history of hyperuricemia
